# Immunofocusing humoral immunity potentiates the functional efficacy of the AnAPN1 malaria transmission-blocking vaccine antigen

**DOI:** 10.1038/s41541-021-00309-4

**Published:** 2021-04-06

**Authors:** Nicole G. Bender, Prachi Khare, Juan Martinez, Rebecca E. Tweedell, Vincent O. Nyasembe, Borja López-Gutiérrez, Abhai Tripathi, Dustin Miller, Timothy Hamerly, Eric M. Vela, Ryan R. Davis, Randall F. Howard, Sandrine Nsango, Ronald R. Cobb, Matthias Harbers, Rhoel R. Dinglasan

**Affiliations:** 1 Emerging Pathogens Institute, Department of Infectious Diseases and Immunology, College of Veterinary Medicine, University of Florida, Gainesville, FL USA; 2 Ology Bioservices, Inc., Alachua, FL USA; 3 Department of Molecular Microbiology & Immunology, Johns Hopkins Malaria Research Institute, Baltimore, MD USA; 4 Center for Global Health, Division of Parasitic Diseases and Malaria, Malaria Research and Reference Reagent Resource Center in the Entomology Branch of the Centers for Disease Control & Prevention, Atlanta, GA USA; 5 Infectious Disease Research Institute, Seattle, WA USA; 6 University of Douala, Faculty of Medicine and Pharmaceutical Sciences, Douala, Cameroon; 7 CellFree Sciences, Co. Ltd, Yokohama, Japan; 8 RIKEN Center for Integrative Medical Sciences, Yokohama, Japan

**Keywords:** Protein vaccines, Infectious diseases, Vaccines, Malaria

## Abstract

Malaria transmission-blocking vaccines (TBVs) prevent the completion of the developmental lifecycle of malarial parasites within the mosquito vector, effectively blocking subsequent infections. The mosquito midgut protein Anopheline alanyl aminopeptidase N (AnAPN1) is the leading, mosquito-based TBV antigen. Structure-function studies identified two Class II epitopes that can induce potent transmission-blocking (T-B) antibodies, informing the design of the next-generation AnAPN1. Here, we functionally screened new immunogens and down-selected to the UF6b construct that has two glycine-linked copies of the T-B epitopes. We then established a process for manufacturing UF6b and evaluated in outbred female CD1 mice the immunogenicity of the preclinical product with the human-safe adjuvant Glucopyranosyl Lipid Adjuvant in a liposomal formulation with saponin QS21 (GLA-LSQ). UF6b:GLA-LSQ effectively immunofocused the humoral response to one of the key T-B epitopes resulting in potent T-B activity, underscoring UF6b as a prime TBV candidate to aid in malaria elimination and eradication efforts.

## Introduction

Malaria was responsible for about 405,000 deaths in 2019, predominantly among children under the age of five in Sub-Saharan Africa^1^. To achieve malaria elimination and eradication, we must leverage concerted approaches to reduce clinical disease and prevent new cases. Vaccines have been considered a major component of the elimination and eradication strategy. However, RTS,S (Mosquirix™), the most advanced malaria vaccine that has been rolled out in selected African countries, reduces clinical disease, but does not prevent new cases. While this vaccine is an important step forward in the fight against malaria, alone it cannot ultimately support malaria eradication since it does not completely prevent malaria transmission^2–4^.

The *Plasmodium* parasites responsible for causing malaria have an obligatory developmental cycle in the female *Anopheles* mosquito vector, which begins when a mosquito takes up male and female *Plasmodium* gametocytes in a blood meal from infected humans. Within the mosquito blood meal bolus, the gametocytes transform to male and female gametes that combine to form motile zygotes (ookinetes), which must first attach to the midgut epithelium followed by enterocyte traversal to form an oocyst just beneath the midgut lamina. Within two weeks, mature oocysts rupture releasing thousands of sporozoites that are passively transported to the salivary glands. Malaria parasite transmission is completed once the infectious mosquito injects salivary fluid containing sporozoites while taking a blood meal from a naïve human host.

**Fig. 1:**
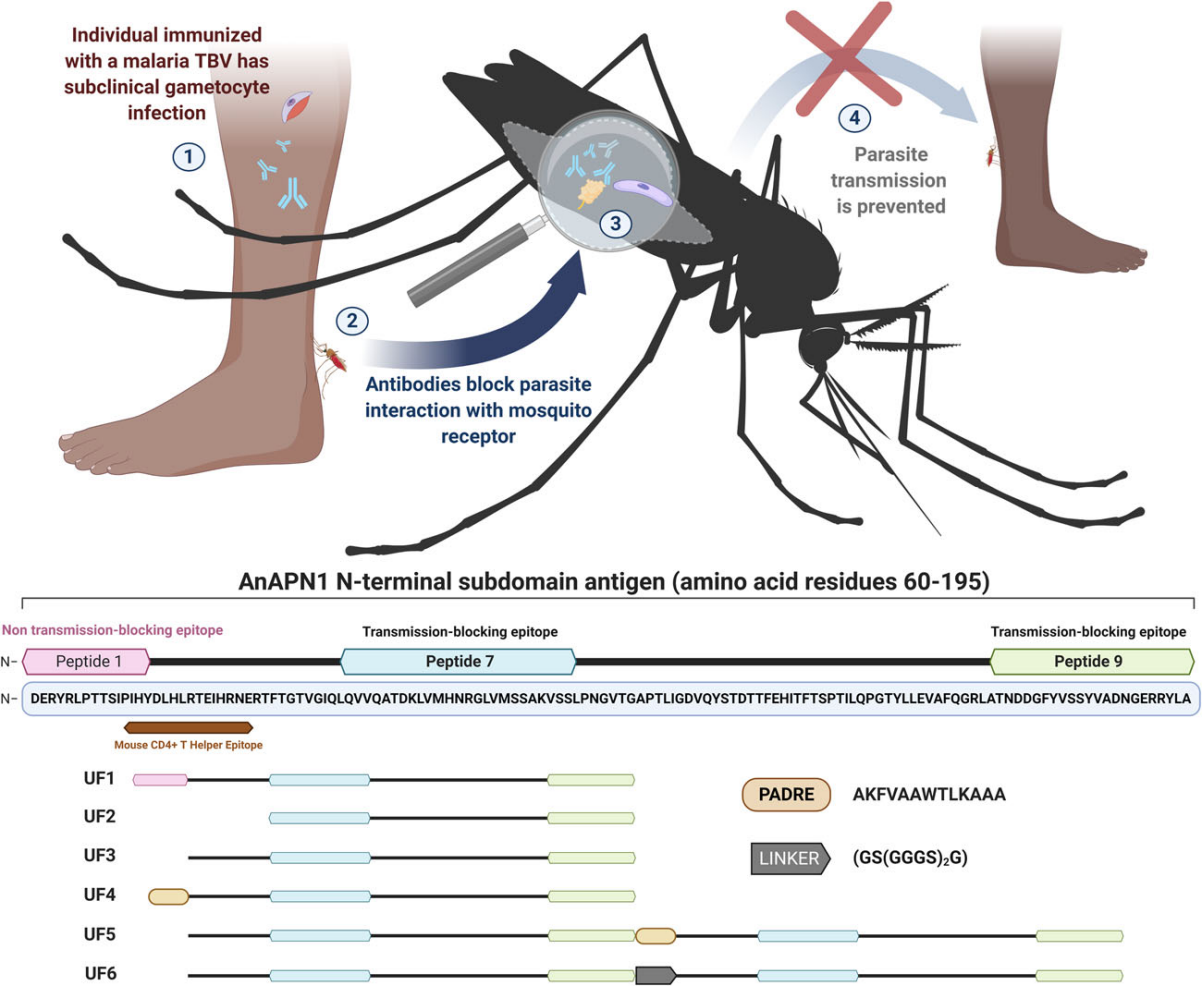
Design of AnAPN1 transmission-blocking vaccine (TBV) constructs. (**Top**) Schematic depicting malaria mosquito-based TBV mode of action, wherein antibodies generated in a human host **➀** are taken up by the mosquito **➁** and prevent parasite (purple)-protein receptor (yellow) interaction **➂** and subsequent development within the mosquito, effectively blocking the spread of parasites within a community **➃**. **(Bottom)** AnAPN1 subdomain antigen design. Several second-generation immunogen constructs of AnAPN1 were developed based on structural and functional information for peptides 1, 7, and 9 (note: peptide numbers were not assigned sequentially from left to right). The location of a Mouse T Helper Epitope (brown) identified through the Epitope Identification Suite (Merck Research Labs) is indicated^7^, which overlaps with the carboxyterminal side of peptide 1. AnAPN1 constructs; UF1: Original AnAPN1 design, UF2: Mouse T Helper Epitope Deletion, UF3: Peptides 2-9 only, UF4: [PADRE: AKFVAAWTLKAAA] + Peptides 2-9 (UF3), UF5: UF3 + PADRE + UF3 construct, UF6: UF3 + [Glycine Linker: GS(GGGS)_2_G] + UF3. (Protein domains are not drawn to scale). See Supplementary Table 1 for additional details.

Transmission-blocking vaccines (TBVs) work by preventing parasite sporogonic development in the mosquito, thereby disrupting the transmission cycle (Fig. 1a)^2–5^. As a result, they also have the added benefit of preventing the spread of parasites that have developed drug resistance^2,6^. As a complementary intervention to RTS,S, TBVs may extend the utility of the RTS,S vaccine by reducing parasite breakthrough transmission in vaccinated populations^2–7^. While several TBVs that target *Plasmodium* proteins have already been tested in the clinic, parasite-centric vaccines are inherently species-specific, requiring the production and validation of several malaria vaccine modalities^2,3^. In contrast, a pan-malaria TBV, which targets a conserved mosquito protein that acts as a parasite receptor in the mosquito midgut, may address the limitations inherent in parasite-centric vaccines.

The mosquito midgut surface protein Anopheline Alanyl aminopeptidase N (AnAPN1) is an advanced, pan-malaria mosquito-based TBV candidate^7^. Antibodies raised against the N-terminal domain of this protein prevent *Plasmodium falciparum* ookinetes from traversing the midgut wall and developing into oocysts, blocking further development and transmission of the parasite^7–10^. Our previous studies demonstrated that anti-AnAPN1 antibodies can completely (100%) block transmission of naturally circulating *P. falciparum* in Cameroon^7–10^. By solving the crystal structure of AnAPN1, we mapped two conformational and protective T-B epitopes, peptides 7 and 9, and a non-protective epitope, peptide 1^7,8^. Peptide 1 includes an immunodominant mouse CD4+ T cell epitope, allowing it to act as an “immune decoy” and elicit a significant humoral response with a robust but non-protective antibody titer^8^. A monoclonal antibody that targets peptide 7 alone^8^, and polyclonal antibodies that target only peptide 9 can both result in potent T-B activity^7^. Therefore, developing a vaccine formulation that promotes a highly focused and functional humoral response to target peptide 7 or peptide 9 while eliminating the non-protective peptide 1 has great potential to be an effective TBV.

To achieve the optimal vaccine formulation, we hypothesized that specific alterations to the immunogen design from the original 135 amino acid subdomain design of the AnAPN1 TBV (135 amino acid recombinant AnAPN1)^7,8^ would focus the humoral immune response to both critical peptide epitopes (peptides 7 and 9) and potentiate T-B activity, thereby allowing lower antibody titers to achieve potent T-B activity. Here, we report the results of an immunological and functional screen in mice of second-generation AnAPN1 constructs that do not contain the mouse immunodominant peptide 1 epitope and are capable of immunofocusing the vertebrate humoral response to critical T-B epitopes. In this study, we not only demonstrated the marked immunogenicity and T-B functionality of the selected second-generation AnAPN1 construct (UF6), but also identified a potent formulation of the preclinical product and established a purification process for a tag-free immunogen (UF6b) with the human-safe Glucopyranosyl Lipid Adjuvant in a liposomal formulation with saponin QS21 (GLA-LSQ) adjuvant^9,11^. Overall, our results highlight UF6b:GLA-LSQ as a strong TBV candidate that may be a critical component in the fight to eliminate if not eradicate malaria.

## Results

### Down-selection to the UF6 immunogen construct

Previous studies on the T-B activity of anti-AnAPN1 IgG together with data on the protein structure revealed that there are multiple epitopes on the AnAPN1 protein; these studies also show that only some epitopes contribute to T-B activity, while others, such as the immunodominant decoy peptide 1, do not^7,8^. Hence, we used these data to inform the design of new immunogen variants to specifically present to the immune system only the relevant T-B domains of AnAPN1, thereby effectively avoiding undesired immunodominant epitopes. Using a wheat-germ cell-free protein expression system, we prepared as a reference standard the original AnAPN1 antigen construct (referred to as UF1 here), along with five new antigen candidates for the screening study (Fig. 1b, Supplementary Fig. 1a, b). A UF2 antigen was constructed based on UF1 without peptide 1, the mouse T helper epitope (RTEIHRNERTFT) and the flanking regions (Fig. 1b); however, UF2 produced insufficient yields in the wheat-germ system, preventing further study. The four other constructs were as follows: UF3 deleted the decoy peptide (peptide 1) and consisted of peptides 2-9; UF4 consisted of a 13 amino acid, non-natural, pan-DR-binding human T helper epitope [PADRE: AKFVAAWTLKAAA] and the UF3 sequence; the UF5 antigen was a dimer construct of UF3 + PADRE + UF3; and finally, the UF6 antigen was a dimer construct similar to UF5 but connected with a glycine linker (GS(GGGS)_2_G) (Fig. 1b, Supplementary Table 1).

After production, we formulated the antigens with either Alhydrogel™ or GLA-LSQ adjuvant and tested them in immunization studies with a prime and two-boost regimen (boosting on days 28 and 42) using outbred CD1 mice. To determine which vaccine formulations were candidates for further development, we ranked the formulations from 1 to 3, with 1 being ‘capable of inducing an antibody response to both peptide 7 and 9’; 2 being ‘capable of inducing an antibody response to either peptide 7 or 9’; and 3 being ‘capable of inducing functional T-B activity’. Our initial immunological screen demonstrated that wheat-germ expressed AnAPN1 constructs UF5 and UF6 were more immunogenic than the original AnAPN1 construct (UF1) (Supplementary Figs. 2, 3). Based on enzyme-linked immunosorbent assay (ELISA) serum endpoint titers alone, the immunogenicity of UF5 appeared superior to that of other constructs. Antibodies generated using UF5:GLA-LSQ or UF6:GLA-LSQ were also found to be focused on the T-B epitope, peptide 9, and the binding specificity appeared greater than that observed for UF1 (Supplementary Fig. 3).

Serum samples from the immunized mice were then used to further determine the T-B activity of the different antigens in both the standard membrane feeding assay (SMFA) using laboratory strains of *Plasmodium* (NF54)^12^ and by direct membrane feeding assay (DMFA)^7–10,13,14^ using circulating parasite strains in Cameroon (Supplementary Fig. 4). Including the wild-type *Plasmodium* strains further demonstrates the high T-B potential of the two “down-selected” antigen candidates (UF5 and UF6) that had also shown the highest endpoint titers in the immunization studies (Supplementary Fig. 2, Supplementary Fig. 3). However, our results indicated that there was no clear correlation between the antibody titer and T-B activity. Although we did not initially observe any advantage in terms of immunogenicity of formulating UF5 with GLA-LSQ over Alhydrogel™ (ALUM) (Supplementary Fig. 3), the subsequent functional studies suggest that the UF6:GLA-LSQ formulation produces the most effective T-B antibodies (Supplementary Fig. 4). Moreover, despite higher UF5:GLA-LSQ antibody titers, the UF6:GLA-LSQ antibodies proved to be more potent at blocking oocyst development in a DMFA (Supplementary Fig. 4).

### Establishing a large-scale cGMP production process for UF6b

**Fig. 2:**
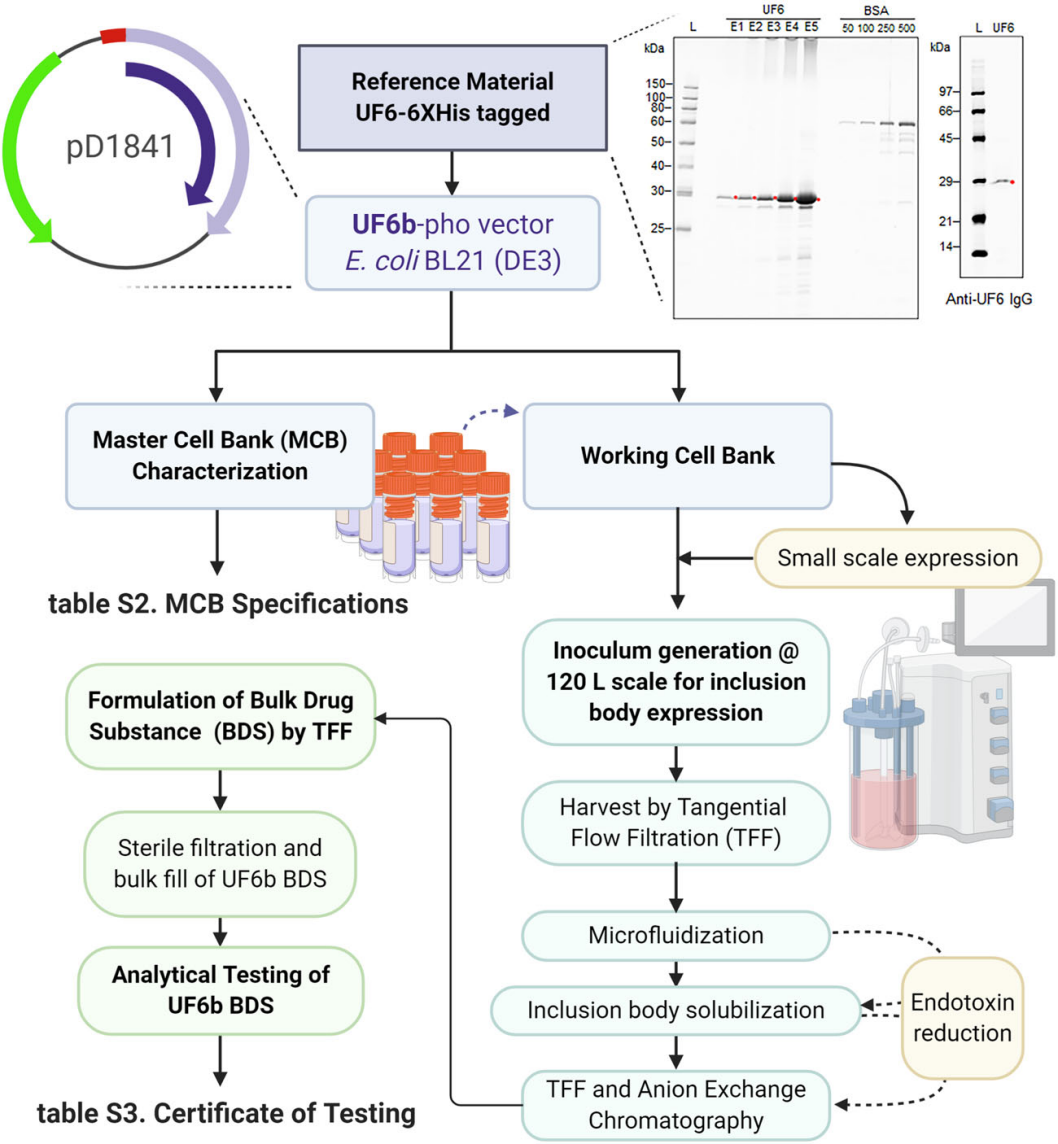
Process flow diagram for UF6b manufacture. (Top right) UF6 antigen produced by CellFree Sciences in the wheat-germ cell-free system, which was recognized as a 29 kDa band by anti-UF6 antibody, was used as reference material by Ology Bioservices for process development. (Top left) *E.coli* BL21 (DE3) was transformed with UF6b-phopD1841 to produce a Master Cell Bank (MCB) expressing the UF6b, His-tag-free antigen. Using a lot from the MCB, pilot small-scale expression was followed by replicate 120-L scale runs with the goal of high-yield production of UF6b in inclusion bodies to aid in the purification steps to reduce endotoxin. In-process UF6b was used for all immunological and functional assays. Bulk Drug Substance was further characterized and observed to yield low endotoxin levels. kDa, kilodalton. BSA, bovine serum albumin standards. See Supplementary Table 2 and Supplementary Table 3 for more details.

Based on the finding that UF6 produced the most robust T-B antibodies, we developed a process to produce large-scale cGMP quantities of the UF6 immunogen in preparation for First-In-Human clinical trials. In this process, we made two changes to the antigen resulting in the creation of the “UF6b” immunogen: (i) removal of the C-terminal His-tag that was used for initial purification and (ii) addition of an inert, non-immunogenic peptide sequence, CGGSG at the C-terminus to protect from anticipated carboxyterminal protease clipping at higher fermentation scales (Supplementary Table 1). This construct was inserted into the *E. coli* expression vector pD1841 for expression under the control of phoA promoter in transformed BL21 (DE3) cells (Fig. 2). A Master Cell Bank was generated and used to produce UF6b (Supplementary Table 2). The identified process produced 52.3 g of total UF6b at harvest (469.94 mg/mL in 111.3 L volume) in relatively low biomass of 45.8 g/L at the 120 L fermenter scale. This calculates to a specific productivity of 1% (0.010 g UF6b per g of *E. coli*). The preclinical grade bulk drug substance met all established acceptance criteria for quality attributes, including an endotoxin level of 6.7 EU/mg (Supplementary Table 3, Supplementary Fig. 5).

### UF6b is immunogenic and elicits anti-peptide 9 antibodies in mice

**Fig. 3:**
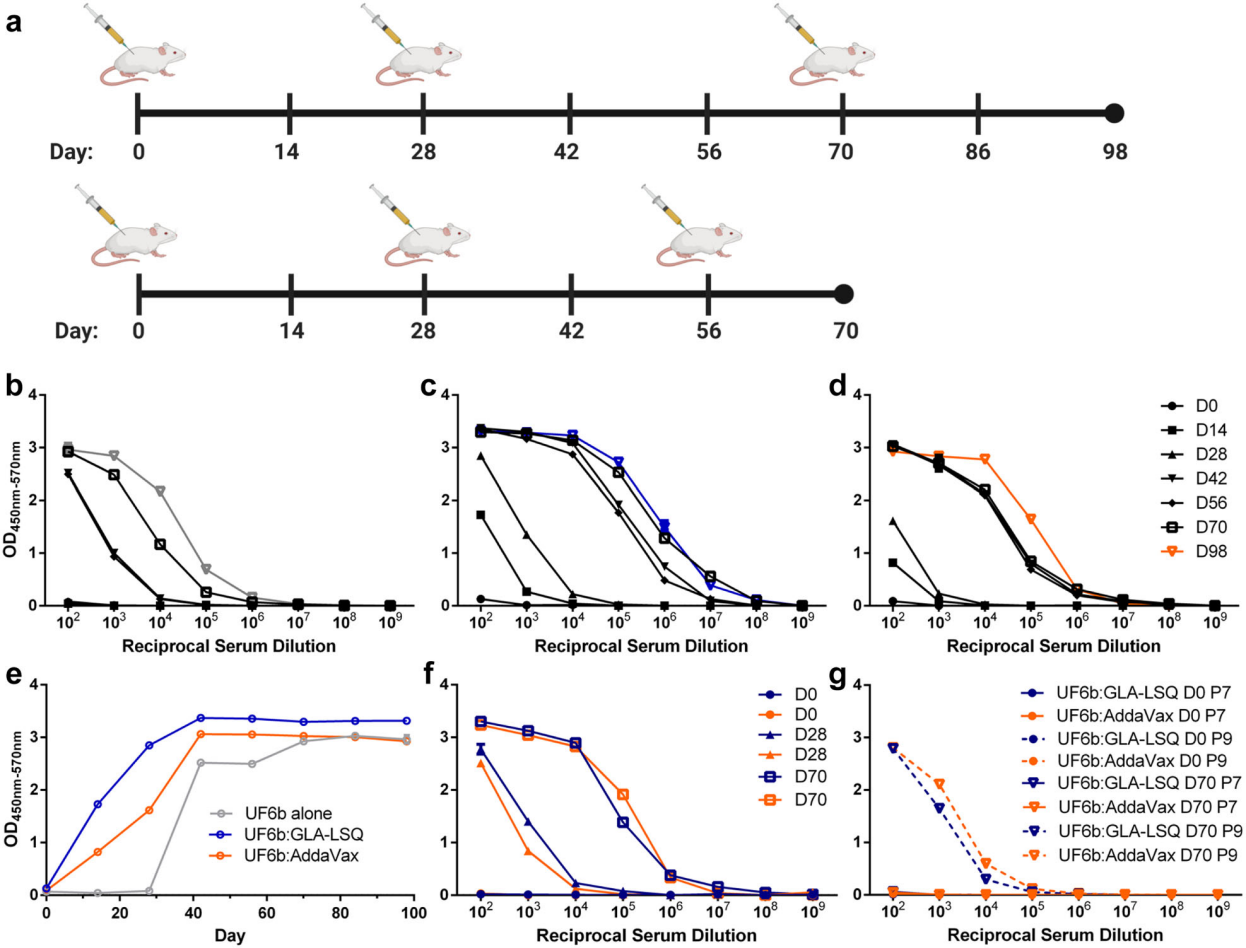
Immunogenicity of the UF6b construct. **a** Mice were immunized (i.m.) following a longer D98 prime and two-boost regimen (boosts on Days 28 and 70) or a compressed D70 prime and two-boost regimen (boosts on Days 28 and 56) (20 μg/dose/mouse). Analyses of the longer regimen sera are shown in (**b**–**e**). **b**–**d** Mice were immunized with UF6b [gray] in the absence of adjuvant (**b**), with UF6b:GLA-LSQ [blue] (**c**), or with UF6b:AddaVax™ [orange] (**d**). **e** The kinetics of the antibody (1:100 dilution) response over time. Analyses of the shorter regimen sera are given in (**f**-**g**): (**f**) UF6b-specific ELISA with sera from mice immunized with UF6b with either GLA-LSQ [blue] or AddaVax™ [orange] in the 70-Day study. **g** Peptide 7 [P7] and peptide 9 [P9] specific ELISA of the same 70-Day study. ELISA analyses used pooled sera collected at the designated timepoints. Absorbances taken at 570 nm were deducted from absorbances at 450 nm to account for background. Error bars indicate SEM of triplicates; however, error was tightly controlled between replicates resulting in compact bars obscured by the data points. D, Day.

To test the immunogenicity of the selected new preclinical UF6b product, outbred CD1 mice were immunized intramuscularly (20 μg/dose/mouse) following a prime plus two-boost regimen, with boosting on Days 28 and 70 to reflect an anticipated dosing interval in a clinical trial (Fig. 3a, Supplementary Table 4). UF6b alone (in the absence of adjuvant) was immunogenic in mice, achieving reciprocal serum endpoint titers of 10^6^ on Day 98 of the study (Fig. 3b), establishing its baseline immunogenicity. Mice were also immunized with UF6b formulated with either GLA-LSQ (Fig. 3c) or AddaVax™ (Fig. 3d). AddaVax™ is the research use only (RUO) version of the more potent MF-59 adjuvant that has been approved for use in humans, and it was used as the positive control to represent a more potent comparator adjuvant than ALUM. The formulation of UF6b:GLA-LSQ elicited a higher, more rapid, and more stable response than either UF6b alone or UF6b:AddaVax™ (Fig. 3e).

Considering that immunization regimens can influence the magnitude and durability of antibody responses to an immunogen, we also tested a Day 0 (prime) with a Day 28 and Day 56-boosting regimen to determine whether a more contracted 70-Day study would affect the overall humoral immune response in mice compared to the longer 98-Day study. We found that the contracted 70-Day immunization regimen (Fig. 3a) produced a comparable antibody response to the 98-Day study with serum endpoint titers at ~1:10^8^ (Fig. 3f).

We hypothesized that deleting the N-terminal “decoy” epitope (peptide 1) would focus the humoral response of immunized mice to the two key T-B epitopes, peptide 7 and peptide 9. We tested this hypothesis by conducting indirect peptide ELISAs using only synthetic peptides for these two epitopes. Using sera collected from the 70-Day study as a representative humoral response for the two different dosing regimens, we observed that the mice mounted an antibody response to peptide 9 but failed to mount a response to peptide 7 when UF6b was formulated with either adjuvant (Fig. 3g). To further confirm these findings, both the longer and contracted dosing regimens were repeated with new cohorts of mice, and the pooled antibody responses generated with UF6b remained similar (Supplementary Fig. 6). Importantly, a closer examination of individual mouse antibody titers from the 70-Day study indicated that the vaccination regimen induced a strong antibody response in each individual mouse (Supplementary Fig. 7).

### UF6b elicits potent T-B antibodies in mice

**Fig. 4:**
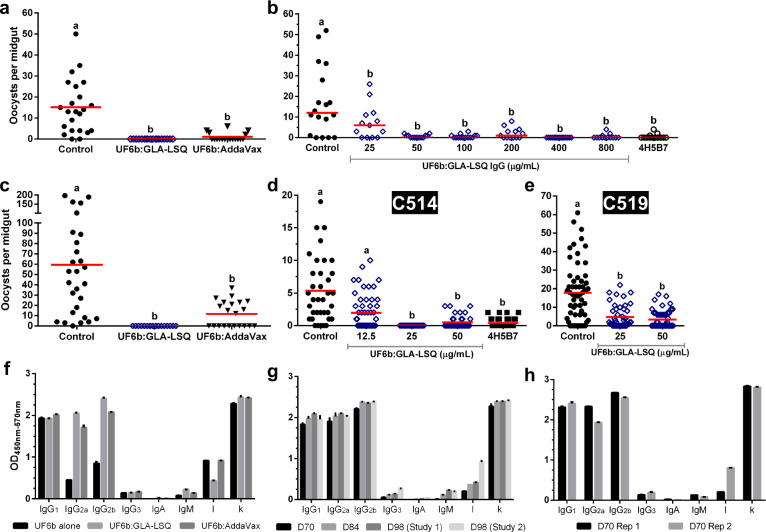
Functional activity of UF6b antibodies. **a**–**e** UF6b:GLA-LSQ generates potent transmission-blocking antibodies against both lab and naturally circulating *Plasmodium* strains. **a** Representative standard membrane feeding assay (SMFA) using 50 µg/mL total IgG purified from pooled sera of mice immunized with UF6b collected on Day 98. **b** Representative SMFA using serial dilutions of purified IgG from Day 70 of the 98-Day study. The monoclonal antibody (mAb) 4H5B7 was included at 250 µg/mL as a positive control. **c** Representative SMFA in the presence of an increased parasite oocyst burden in mosquitoes (*X̄* = 60) in the control group as compared to a representative lower midgut oocyst burden as shown for panel (**a**) controls (*X̄* = 15) using 50 µg/mL total IgG purified from pooled sera of mice immunized with UF6b collected on Day 98. **d**-**e** Direct membrane feeding assay (DMFA) in Cameroon conducted with blood from 2 gametocytemic children (Case codes C514 and C519) with varying concentrations of total IgG. The peptide 7 mAb 4H5B7 served as the positive control. **a**–**e** Total IgG was purified from the same cohort of mice. Treatments without a common letter were found to be statistically significant (α = 0.05, *p* < 0.01, *N* = 14–59) as calculated by Kruskal–Wallis test with Dunn’s post hoc correction or by zero-inflated Generalized Linear Mixed Model as appropriate. Red horizontal bar indicates mean oocyst number per mosquito midgut. Mosquito sample sizes for each control/test group from left to right: (**a**) 39, 43, 46 (**b**) 18, 13, 13, 14, 13, 16, 13, 13 (**c**) 23, 18, 18 (**d**) 36, 54, 44, 40, 48 (**e**) 58, 48, 50. **f** Comparison of Ig isotypes present in antisera from the 98-Day study between UF6b alone, UF6b:GLA-LSQ and UF6b:AddaVax™. **g** Comparison of Ig isotypes from collections at D70, D84, and D98 from the 98-Day studies. **h** Ig isotypes for two replicates of the 70-Day studies. **f**–**h** Sera used at 1:1,000 dilution. Error bars indicate the SEM for replicate wells. See text for details.

Previously, we have shown that immunizing mice with the N-terminal 135-aa recombinant AnAPN1 protein generates functional T-B antibodies^7–10^. In the current study, we assessed whether total IgG purified from mice immunized with the new construct UF6b similarly blocked parasite transmission by both SMFA and DMFA. Antibody titer measurements of purified antigen-specific IgG showed that 50 µg/mL of total IgG contained about 3.3 µg/mL of UF6b antigen-specific IgG. Using 50 µg/mL of total IgG effectively blocked transmission of *P. falciparum* in *Anopheles gambiae* mosquitoes (Fig. 4a–e), leading to a significant reduction in midgut oocyst intensity (i.e., the number of oocysts present per midgut). Antibodies raised in mice immunized with UF6b:GLA-LSQ were able to significantly reduce mean oocyst intensity by more than 90% in treatment groups (*P* < 0.001, GLMM, Kruskal–Wallis, α < 0.05) (Fig. 4a, c). Additionally, higher concentrations of total IgG did not appear to enhance T-B activity at concentrations above 50 µg/mL (Fig. 4b). This phenomenon has been described previously, wherein higher antibody concentrations that either resulted in plateauing of T-B activity or enhancement of parasite infection of the midgut, which are both effects within the known intrinsic error of the assay^13,15–18^.

We also tested the ability of purified total IgG from mice immunized with UF6b:GLA-LSQ to block naturally circulating *P. falciparum* parasites from infecting *An. gambiae* mosquitoes in Cameroon by DMFA. *P*. *falciparum* gametocyte-positive blood was isolated from infected children in Cameroon and UF6b-specific IgG was added to the blood in each membrane feeder and used to feed to mosquitoes. UF6b-specific IgG at 3.3 µg/mL resulted in reproducible reductions of 80–90% in mean oocyst intensity (*P* < 0.001, GLMM, α < 0.05) (Fig. 4d, e).

To determine whether the observed differences in apparent T-B activity for antisera from UF6b:GLA-LSQ and UF6b:AddaVax™ immunizations could be explained by variances in antibody quality and/or quantity, we compared the antigen-specific immunoglobulin isotypes for all groups in both the 98-Day and 70-Day dosing studies. In the absence of adjuvant, UF6b elicited primarily an IgG_1_ response with lower levels of IgG_2a_ and IgG_2b_ (Fig. 4f). When given in conjunction with either GLA-LSQ or AddaVax™, the immunoglobulin profile was more equally divided between these three subtypes. Also, between these three immunization groups no differences were noted for IgG_3_, IgA, and IgM, which were all comparably low, and all groups had a higher proportion of kappa to lambda light chains (Fig. 4f). Very little variation in the immunoglobulin profile was seen in the 98-Day study after the D70 timepoint, although we did notice an increase in lambda light chain in the repeat 98-Day study (Fig. 4g). We noted that the 70-Day study of UF6b:GLA-LSQ produced a comparable immunoglobulin subclass profile (Fig. 4h) as compared to the longer 98-Day study (Fig. 4g), indicating that flexibility in dose regimen schedules could be used in future clinical studies without affecting the antibodies produced. There was some variation in the amounts of IgG_2a_ and lambda light chain between the two replicate 70-Day studies, which can be expected with outbred mice.

## Discussion

Here we successfully optimized the mosquito-based AnAPN1 vaccine candidate for further development and evaluation as a potent malaria TBV antigen. Moreover, our studies further demonstrated the benefits of using structural information in antigen optimization as a crucial step in the development of any vaccine candidate. Higher quality constructs were formed based on a structure-function understanding of the immunogenic and mosquito-specific domains of the full-length APN1 from *An. gambiae*^10^. We showed that presenting the key T-B epitopes twice in dimer constructs, such as the design of UF5 and UF6, increased the overall immunogenicity and T-B activity compared to the original UF1 construct. These two constructs also successfully focused the humoral response to the key epitope peptide 9, although UF6 ultimately proved most effective in the functional studies and was chosen for further development. The preclinical product UF6b was reliably immunogenic in mice even when inoculated alone without adjuvant. UF6b generated a peptide 9-specific response, which we have demonstrated previously^8^ confers potent T-B activity. While our original goal had been to target both peptides 7 and 9, which were predicted to strongly bind MHC II encoded by the HLA-DRB1 alleles common to East African populations^7,11^, we were unable to elicit a peptide response specific to peptide 7. However, the lack of a peptide 7-specific response did not appear to affect the overall T-B activity of the antibodies. Overall, the UF6b TBV candidate designed and assessed here achieves two priority goals: induction of antibodies to either peptide 7 or peptide 9 and demonstration of functional T-B activity at lower antibody concentrations.

In previous studies, between 0.8 and 1.6 mg/mL of purified total IgG from rabbits and mice immunized with UF1 was needed to block oocyst development in both SMFAs and DMFAs^7^. At this high total IgG concentration, it was estimated that only ~5–10 µg/mL were antigen-specific IgG. However, these studies used an immunogen containing the immunodominant “decoy” epitope peptide 1, causing a larger proportion of the murine IgG profile to target this malaria-irrelevant epitope^7,8^. We observed that a comparable amount of UF6b-specific IgG (3.3 µg/mL) was present in the antisera of mice immunized with UF6b:GLA-LSQ. These data suggest that the UF6b:GLA-LSQ formulation was indeed able to focus or skew the vertebrate immune response towards the peptide 9 epitope, resulting in a 32-fold lower concentration of total IgG (50 µg/mL as opposed to 1.6 mg/mL) needed to confer marked T-B activity. It remains to be seen if antibodies with similar potency can be induced in non-human primates (NHPs) or humans. Maximal T-B activity for total IgG following UF6b:GLA-LSQ immunization was comparable to that observed for the potent monoclonal antibody (mAb) 4H5B7, which targets peptide 7 only^8^. The degree of blocking activity appears to be related to the intensity of the infection of the mosquito in SMFAs and in DMFAs, and the variation in T-B activity may be related to the genetic complexity of the gametocytes in the blood obtained from infected volunteers^19^. This is not entirely unexpected since the membrane feeder (the gold standard for measuring T-B activity in the field) is artificial and circulation of infected red blood cells and antibody within the small feeders is not currently possible. As such, the distribution of antibody throughout the blood meal may not be even. Despite the intrinsic difficulties of ensuring consistent antibody delivery during membrane feeding assays^15–17^, when oocyst intensities were less than 50 oocysts/midgut in control mosquitoes, we observed 100% inhibition in mosquitoes fed with blood containing the anti-sera from immunized mice. These findings are promising, as this level of infection is what would typically be expected in natural infections^20^.

Differences in T-B activity between the UF6b:GLA-LSQ and UF6b:AddaVax™ vaccine formulations cannot be explained by total antibody titers alone. Previous studies suggest that a predominantly Th2 response is elicited in mice following immunization with AnAPN1 (UF1) formulated with Alhydrogel™^10^, which is characterized by lower IgG_2_ titers relative to IgG_1_ titers in immune sera. However, higher IgG_2a_ and IgG_2b_ titers in response to UF6b were observed with UF6b:GLA-LSQ and UF6b:AddaVax™ compared with UF6b alone, indicating a Th1-biased humoral response in these outbred CD1 mice. UF6b:GLA-LSQ elicited a slightly higher IgG_2a_ titer as compared to UF6b:AddaVax™ and more than a log higher IgG_2a_ titer than UF6b alone. Saponin-based adjuvants (GLA-LSQ) and emulsions such as AddaVax™ or MF-59 are designed to induce robust and balanced Th1/Th2 responses, and this was clearly observed in our study for UF6b:GLA-LSQ and UF6b:AddaVax™^21^. The observation of a higher proportion of kappa light chains in antigen-specific IgG (all Ig subclasses) for the UF6b:GLA-LSQ has not been previously observed in studies using UF1:Alhydrogel™. In humans, variable light chains can influence antibody-binding specificity, serum half-life, and conformational flexibility in addition to other physicochemical properties^22^. IgG_3_ responses have not been noted in our previous work with AnAPN1-targeted immunization, and although associated with a Th1 profile, the overall IgG_3_ titers in this study were low. Given that the potent mAb 4H5B7 is an IgG_2b_, kappa isotype, it is possible that the higher titers of IgG_2a_ and IgG_2b_ and light chain isotype ratio may have resulted in not only more antibody, but also a qualitatively better antibody repertoire. This hypothesis bears further study and could be relevantly and appropriately addressed in a Phase 1 clinical trial. It should be noted that mice are an imperfect model organism for predicting the success of malaria vaccines in humans, as mice generate different immune responses with distinct antibody characteristics as compared to NHPs and humans^23^. As such, further testing of the tolerance, immunogenicity, and longevity of our UF6b:GLA-LSQ formulation are currently being completed in NHPs to allow a quicker transition to First-In-Human clinical trials.

The process development workflow (Fig. 2) established in this study positions the program well to move forward to cGMP production of UF6b. Based on a theoretical dose 0.1 mg/mL/subject as the highest dose in a Phase 1 study, the conservative estimated endotoxin level would be 0.35 EU/mL. This is already well below the <20 EU/mL recommendation for each dose of a recombinant protein vaccine and is 15-fold lower than the upper limit of endotoxin for Rubella vaccines^24^. In fact, the current process sets the AnAPN1 (UF6b) endotoxin level within the reported range of several recommended pediatric vaccines^24^.

Malaria TBVs are a critical component in the fight to eliminate and eradicate malaria. Our results meet and surpass the functional benchmark criteria for a successful TBV, which are that antibodies against a TBV candidate should block ≥80% of *Plasmodium* oocyst development and achieve this level of activity using lower concentrations of target-specific antibody in the membrane feeding assay, as has been described previously^5^. Considering the inherent variation in vaccine responses in human populations in malaria-endemic countries, partly because of acute malaria, concomitant infections and/or malnutrition^25^, it is important that the concentration of antibodies required for functional malaria T-B activity be reduced as much as possible. Doing so will increase the likelihood that implementing such a vaccine would result in demonstrable efficacy at the community level. The use of the GLA-LSQ adjuvant is predicted to help overcome limitations in the induced T follicular helper (Tfh) cell response, as circulating Tfh cells were found to be impaired in B cell help during an acute malaria episode^25^, and this adjuvant has proved to be critical in improving the total Tfh response in preclinical studies with the Pfs25 TBV^26^. Overall, our results are promising and underscore the candidacy of the AnAPN1 molecule (exemplified by UF6b:GLA-LSQ) as a prime malaria TBV formulation. Coupled with the high-yield and low endotoxin level of the current preclinical Drug Product, the AnAPN1 TBV is positioned nicely to move forward with cGMP manufacture, lot release testing, and toxicity studies as a prelude to a Phase 1 clinical trial.

## Methods

### Experimental design

For both SMFAs and DMFAs, IgG was purified from each 0.8-1 mL cardiac puncture sample per mouse. Since a single mouse cannot yield sufficient purified IgG for an assay, using current best practices, immune serum from either ten or twenty mice was pooled before purification. The purified IgG from the pooled immune sera permitted us to perform dose-response membrane feeding assays in triplicate.

Data collection was stopped at a predetermined timepoint of either Day 70 or 98. As per the animal care use committee approved protocol (protocol #201909359), data collection endpoints were also determined by the following humane endpoints for the test mice: if any of the test mice sustained more than or equal to 15% weight loss from baseline weight or age-matched controls for those animals still maintained during the study; a body condition score of 2 or less; inability to reach food or water; impaired mobility; the presence of tumors; labored breathing, respiratory distress or cyanosis (blue tinged color); tremors, convulsions or seizures lasting more than 1 min or occurring more than once a day; dehydration lasting over 24 h that is unresponsive to treatment; or moribund - unable to right itself.

The objective of this study is to down-select to a new AnAPN1 construct capable of immunofocusing the humoral response to the two key T-B epitopes. Once this was accomplished, we sought to determine the immunogenicity and immunofocusing ability of the UF6b antigen as well as quantify the T-B activity of the elicited immune sera. To test if UF6b immunofocused the murine humoral response to peptides 7 and 9, mice were immunized via intramuscular injection (i.m.) with either UF6b alone, UF6b:GLA-LSQ, or UF6b:AddaVax™. At pre-specified timepoints blood samples were collected to perform ELISAs, SMFAs, and DMFAs. All relevant data are included. The study was performed twice per dosing regimen with each mouse serving as a biological replicate in each cohort. Tests were performed with two or three technical replicates. There was no blinding in this study.

### Ethics

All research protocols have been approved by the relevant institutional review boards and national ethics committees for Cameroon (2016/03/730 and 2019/07/1168/CE/CNERSH/SP) and the University of Florida (UF) for concurrence and laboratory-based studies (IRB201800971, IRB201902261, and IRB201602150). Informed consent was obtained from all participants. The use of vertebrate animals (mice) for these studies have been approved by the UF Animal Care and Use Committee (UF Animal Welfare Assurance number A3377-01, Protocol# 201909359, approved on 06/07/2019).

### Wheat-germ cell-free immunogen expression

Templates for the expression of proteins UF1 to UF6 were prepared by gene synthesis (GenScript, USA). Before synthesis, sequences were optimized for expression in wheat, and a His-tag with six histidine residues was directly added without spacer to the C-terminus of the proteins. Templates were then cloned into the expression vector pEU-E01-MCS (CellFree Sciences, Japan) using the XhoI and BamHI restriction sites, and the DNA sequences of the templates confirmed before use in protein production. Plasmid DNA for each template was prepared using a Plasmid Maxi Kit (Qiagen, Germany). Protein expression was first confirmed by performing a 50 µL dialysis expression reaction using the wheat-germ cell-free protein expression system extract WEPRO7240H (CellFree Sciences, Japan) according to the manufacturer’s directions for performing separate transcription and translation reactions. After a 72-h translation reaction, the crude reaction mixtures were centrifuged for 10 min at 21,500 × *g* to remove the insoluble fraction before loading onto a chelated-Ni resin (Ni-NTA Agarose, Qiagen, Germany). Proteins were purified on the Ni resin using a commercial buffer set (His Buffer Kit, GE Healthcare, USA). In brief, crude reaction mixtures were centrifuged for 10 min at 21,500 × *g* to separate the soluble from the insoluble fraction. The samples were diluted 4-fold using a buffer containing 20 mM Na-phosphate, 0.5 M NaCl, 0.02 M imidazole (pH 7.5) before loading onto the resin. After the binding reaction, the resins were washed three times with a 20 mM Na-phosphate, 0.5 M NaCl, 0.05 M imidazole buffer before the proteins were eluted twice with 20 mM Na-phosphate, 0.5 M NaCl, 0.5 M imidazole Elution Buffer. Proteins from each fraction during expression and later processing were resolved by 15% SDS-PAGE (DRC, Japan) and stained with Coomassie Brilliant Blue. Different amounts of BSA were run in parallel to quantitate yields of the expressed proteins. Because protein UF2 was partially insoluble and expressed at lower yields compared to the UF1 protein, further studies with UF2 expression were discontinued in favor of UF1. The remaining proteins UF1 and UF3 to UF6 were produced on a larger scale using 3 mL dialysis translation reactions following the same procedures established during the expression tests. As a part of large-scale protein preparation, a buffer containing 15% sucrose, 10 mM Tris, 0.2% Tween-80 (pH 7.8) replaced the Elution Buffer. Proteins were shock-frozen in liquid nitrogen and stored at −80 °C until later use.

### Process development of UF6b manufacture

UF6 sequence was modified to remove the C-terminus His-tag and in its place, we added a protective C-terminal, inert peptide linker sequence (CGGSG) to generate UF6b^27^. The produced sequence (GenScript) was cloned into the expression vector pD1841 under phoA promoter control and kanamycin as a selection marker. Competent *E. coli* BL21(DE3) (ThermoFisher) were transformed and screened for expression of UF6b by Western blot. Research cell banks (RCB) and Master cell bank (MCB) were generated for the production of UF6b. Process development was performed in scale-down models in a 10 L glass-vessel bioreactor (BioFlo 320, Eppendorf). Established process parameters were then scaled up to 120 L in a BioFlo Pro 150 stainless steel fermenter (Eppendorf). Phosphate depletion during the growth phase induced UF6b expression into inclusion bodies. Harvest was performed via tangential flow filtration using a 750 kDa hollow fiber cartridge (Spectrum/Repligen) at load challenge <50 g/m^2^. The biomass was concentrated to >100 g/L and buffer exchanged into 50 mM HEPES (pH 8.0) buffer and subsequently diluted to 50 g/L for mechanical lysis by microfluidization (Microfluidics M-110E) at 20,000 psi for four (4) cycles with temperature controlled at ≤16 °C. The insoluble inclusion body (IB) fraction was separated by centrifugation at 10,000 × *g* for 30 min at 2–8 °C (Sorvall Lynx 6000, ThermoFisher). The IB pellet was washed with 20 mM HEPES (pH 8.0), 2 M urea, 1% Triton X-100 at a 1:10 ratio (g of WCW biomass to wash buffer) and isolated by centrifugation. Two-phase separation wash with Triton X-114 was performed to reduce endotoxin levels^28^. The washed pellet was then solubilized with 6 M urea in 20 mM HEPES (pH 8.0) buffer for 1 h with mild agitation, and an additional endotoxin removal step was performed on the soluble preparation as previously referenced. Solubilized UF6b was refolded by dilution (from 6 M urea to 1 M urea) with 20 mM HEPES (pH 8.0) and buffer exchanged into 20 mM Tris-HCl (pH 8.0) + 15% sucrose + 200 mM NaCl by TFF to prepare the material for further purification by anion exchange chromatography (AEX).

UF6b polishing was performed by tandem AEX on Capto Q ImpRes (Cytiva) medium at a linear velocity of 150 cm/h in flow-through mode followed by bind and elute mode. The effluent from flow-through mode was diluted with 20 mM Tris-HCl (pH 8.0) to achieve conductivity <7 mS/cm. After equilibration with 20 mM Tris-HCl (pH 8.0), binding of UF6b to AEX at ≤50 mg/mL resin was followed by a 5-column volume (CV) wash with equilibration buffer and elution by linear gradient from 0 to 500 mM NaCl over 15 CV. Peak fractions were further analyzed for impurities (host cell DNA, host cell protein), overall purity, and endotoxin content and then pooled. Formulation was performed by TFF (ProStream mPES,1 m^2^ 10 kDa MWCO, L-screen; Repligen) operated with a TMP of 15 psig at a process flux of 8 LMH. The retentate was concentrated to >1 mg/mL then buffer exchanged with 7 diavolumes of 20 mM HEPES, 15% sucrose, 0.2% Tween 20 (pH 8.0). After the retentate was recovered, Tween-20 was added to Bulk Drug Substance (BDS) to a final concentration of 0.2%, and the BDS was filtered through a sterilizing grade 0.2 µm filter (Opticap PES, Millipore Sigma) to generate formulated BDS. BDS was aliquoted into HDPE bottles and stored at ≤−65 °C.

### Mouse immunizations

To down-select to the optimal AnAPN1 construct, female outbred CD1 mice (*N* = 5) aged 5–7 weeks (Charles River) were immunized with 20 μg of either wheat-germ cell-free expressed UF1, UF3, UF4, UF5, or UF6 emulsified 1:1 with GLA-LSQ or Alhydrogel™. Mice were primed subcutaneously and boosted on Days 28 and 42 intraperitoneally (i.p.) for antigens formulated with Alhydrogel™ and intramuscular (i.m.) for antigens formulated with GLA-LSQ. Sera was collected on Day 0 and prior to boosting on Days 28 and 42. On Day 56 the mice were sacrificed, and sera collected via cardiac puncture.

To test the new UF6b construct, female CD1 outbred mice (Charles River) aged 6–8 weeks were used. Forty mice were split into three groups (Supplementary Table 4): Group A contained 10 mice immunized (i.m.) with UF6b; Group B (main focus group) contained 20 mice immunized (i.m.) with UF6b:GLA-LSQ; and Group C contained 10 mice immunized (i.m.) with UF6b:AddaVax™. For all treatment groups, mice received 20 μg UF6b in diluent (20 mM HEPES, 15% sucrose, 2% Tween-20, pH 8.0) to 100 μL total volume without or with adjuvant. For treatment groups B and C described above, 50 μL adjuvant was mixed 1:1 (vol/vol) with 50 μL antigen in diluent (total volume = 100 μL). At Day 0, mice received an i.m. injection of 50 μL of the antigen/adjuvant formulation in each caudal thigh, resulting in a total priming dose volume of 100 μL. At Days 28 and 70 post-priming, all mice were boosted i.m. with the same formulations described above (Fig. 3a). Every two weeks post-priming, mice were bled to collect sera for anti-AnAPN1 antibody titer determination via ELISA. Mice were sacrificed 4 weeks after the final boost and serum was collected via cardiac puncture. A single replicate study was completed with 10 mice immunized with the same UF6b:GLA-LSQ formulation in the same manner and timepoints. Unfortunately, due to COVID-19 closures, Day 84 sera could not be collected.

Two replicate studies with a contracted dosing schedule were completed with 20 female CD1 mice (Charles River) at 6 to 8 weeks of age. Ten mice were immunized with the same UF6b:GLA-LSQ formulation described above, and ten mice received the UF6b:AddaVax™ formulation. All mice received a prime dose i.m. on day 0 and were subsequently boosted on Days 28 and 56 post-priming (Fig. 3a). Sera was collected every two weeks post-priming until the endpoint at Day 70 when the mice were sacrificed.

### Indirect Enzyme-Linked Immunosorbent Assays

Maxisorp 96-well ELISA plates (Nunc, Fisher Scientific) were incubated overnight at 4 °C with 1 μg/mL antigen in 1x PBS (pH 7.2). After three washes with PBS-Tween 20 (0.1%) (PBST20), the plates were blocked for 1 h at room temperature with 1% bovine serum albumin (BSA) in 1xPBS. Serum samples were diluted to 1:50 and 1:100, then diluted ten-fold to 1:10^8^ in 0.5% BSA in 1xPBS. After discarding the BSA and drying the plates, 100 μL of each of the eight serum dilutions were added to each well in triplicate and incubated 1 h at room temperature. Plates were again washed three times with PBST20. For detection, 100 μL of a horseradish peroxidase (HRP)-conjugated goat anti-mouse IgG(H + L) (KPL) diluted 1:5,000 in 0.5% BSA was added to each well and incubated for 1 h at room temperature. Plates were washed three times with PBST20 and developed by adding 100 μL of (3,3′,5,5′-tetramethylbenzidine) microwell peroxidase substrate (KPL) to each well. Development was stopped after 5 min by the addition of 100 μL of 1 M phosphoric acid. A Synergy™ HTX Multi-Mode Microplate Reader was used to read the OD values at 450 nm and 570 nm. The OD value at 570 nm was subtracted from the OD value at 450 nm to correct for background. Serum endpoint titers were defined as the reciprocal serum dilution giving a higher density reading than the average OD value at 450 nm-570 nm of the pre-immune serum plus three standard deviations.

### Peptide indirect enzyme-linked immunosorbent assays

Maxisorp 96-well ELISA plates (Nunc, Fisher Scientific) were coated with 100 μL of Poly-l-Lysine (PLL) at a concentration of 50 μg/mL in 0.05 M sodium bicarbonate buffer (pH 9.6) and incubated covered for 1 h at room temperature. The plates were washed once with PBST20, then coated in 100 μL 1% (v/v) glutaraldehyde in PBS and incubated covered for 15 min at room temperature. The plates were washed again once with PBST20, and then coated with 100 μL of either peptide 7 or peptide 9 (7,8) and incubated covered overnight at 4 °C. The next day the plates were washed twice with PBST20, then 200 μL 1 M glycine was added to each well and plates were incubated covered for 1 h. The plates were washed twice with PBST20 and 300 μL of a 1:1 solution of 5% milk and 1% gelatin was added to the plates and incubated covered for 1 h. The serum samples were diluted in PBS using serial ten-fold dilutions from 1:10^2^ to 1:10^9^. After washing the plates once with PBST20, each of the eight sample dilutions was added to each well in triplicate and incubated covered for 1 h. The plates were washed three times with PBST20 and 100 μL (HRP)-conjugated goat anti-mouse IgG(H + L) (KPL) (ThermoFisher) diluted 1:5000 in PBS was added to each well and incubated covered for 1 h at room temperature. Plates were washed three times with PBST20 and developed by adding 100 μL of (3,3′,5,5′-tetramethylbenzidine) microwell peroxidase substrate (KPL) to each well. Development was stopped after 5 min by the addition of 100 μL of 1 M phosphoric acid. A Synergy™ HTX Multi-Mode Microplate Reader was used to read the OD values at 450 nm and 570 nm. The OD value at 570 nm was subtracted from the OD value at 450 nm to correct for background. Serum endpoint titers were defined as the reciprocal serum dilution giving a higher density reading than the average OD value at 450 nm-570 nm of the pre-immune serum plus three standard deviations.

### Mosquito colony

*An. gambiae* (Keele) was used in SMFAs. Mosquitoes were reared under standard insectary conditions of 27 °C, 80% relative humidity, and 12 h light:12 h dark cycle. Eggs were hatched in milli-Q water supplemented with 0.02% yeast slurry. The emerging larvae were reared in plastic trays (25 cm long × 20 cm wide × 14 cm high) at a density of 300-400 larvae per tray and provided a daily ration of 1 g koi fish food per tray/day. Pupae were collected and put in holding cages for emergence. Emerged adults were fed ad libtum on 10% sucrose solution. For all experiments, 5-to-6-day old adult females were used.

### Standard Membrane Feeding Assays

Briefly, cultures were seeded at 0.5% parasitemia and grown under hypoxic conditions in RPMI culture media supplemented with HEPES, hypoxanthine, glutamine, and 10% heat-inactivated human serum. The RPMI media was changed daily during culturing. Gametocyte cultures were harvested 17-18 days after initiation, and infected red blood cells were brought up in heat-inactivated human serum (LifeSouth Community Blood Center Inc, Gainesville, FL) plus human RBCs (blood type O-positive) at 1% gametocytemia and 30% hematocrit. Infective blood was mixed with control IgG (purified from naïve mouse serum) or total IgG purified from pooled sera of mice immunized with UF6b prior to delivery directly into water-jacketed membrane feeders maintained at 37 °C via a circulating water bath. The final concentration of total IgG in 300 µL total volume of infective blood was 50 µg/mL for each technical replicate. Female *An. gambiae* were starved for 6 h, placed in paper cups with tops covered with netting (*N* = 40 per cup) and allowed to feed from each feeder for 20 min. Fully engorged female mosquitoes were maintained for 8 d when they were dissected for midgut oocyst enumeration. The dissected midguts were stained with 0.1% mercurochrome to visualize oocysts. A total of three biological replicate experiments were conducted.

### Direct membrane feeding assays

Mosquito infections were conducted during the rainy seasons in 2019. Procedures for gametocyte carrier detection, blood collection, and mosquito infections were performed as follows. Briefly, *P. falciparum* gametocyte carriers were identified among asymptomatic children (ages 5 to 11) in primary schools in the Mfou district, 30 km from Yaoundé, Cameroon. Venous blood was drawn from gametocyte-positive individuals (>20 gametocytes/µL of blood) in heparinized Vacutainer tubes from the antecubital fossa. Membrane feedings (500 µL) were set using donor’s blood with the replacement of the serum by a non-immune AB serum. The local laboratory strain of *An. coluzzii*, named Ngousso, was used for mosquito feedings. Following infection, mosquitoes were maintained at the laboratory under standard insectary conditions (27 ± 2 °C, 85 ± 5% RH, 12 h light/dark) for 8 days until dissections. Oocyst enumeration was performed as in the SMFAs described above.

### IgG purification and antigen-specific IgG quantification

Total IgG was purified from pooled serum using Protein A/G Magnetic Agarose Beads (Thermo Fisher Scientific) according to the manufacturer’s instructions. The antibodies were quantified using the Pierce™ BCA protein assay kit. Antigen-specific IgG was purified from pooled serum with UF6b-conjugated Dynabeads™ M-270 Epoxy (Thermo Fisher Scientific) following the manufacturer’s instructions. The antibody titer of the purified UF6b-specific IgG and total IgG (serum) was determined with the Easy-Titer™ Mouse IgG assay kit (Thermo Fisher Scientific).

### Antibody Isotyping

Anti-UF6b immunoglobulin isotypes were determined with the Mouse Typer Isotyping Panel (Bio-Rad). Plates were coated and blocked, as described above, and mouse sera diluted 1:1,000 was added. After washing five times the isotyping panel was added, followed by five washes, then (HRP)-conjugated goat anti-rabbit IgG(H + L) (KPL) (Bio-Rad). Absorbances were detected as described above.

### Statistical analysis

To determine differences in oocyst intensity (defined by the mean oocyst count) between pre-immune sera and test sera in SMFAs the data was first cleaned of outliers using a robust nonlinear regression test (ROUT) and a Q coefficient of 1% for all treatment and control groups^29^ using GraphPad Prism 6. After comparing the raw and cleaned data and considering that assumptions and overall significance results were not affected, outliers were removed to account for the intrinsic noise in the feeding assays, contributed in part by the uneven distribution of spiked antibody and pooling of gametocytes in the membrane feeder as well as variability in mosquito feeding voracity. The cleaned data was then analyzed by either a zero-inflated or normal Generalized Linear Mixed Model statistical test (GLMM) using the *R* package^30^, and when neither was appropriate, a non-parametric Kruskal–Wallis test followed by Dunn’s post hoc for repeated measures test using GraphPad Prism 6^7,8,10,31^.

### Reporting summary

Further information on research design is available in the Nature Research Reporting Summary linked to this article.

## Supplementary information

Reporting Summary

Supplemental Information

## Data Availability

The datasets generated during the current study are available on reasonable request from the corresponding author. The materials described in this study are intended for further malaria vaccine development. Non-cGMP materials can be provided following a request from the authors on a limited use for research purposes only. There are anticipated costs associated with access to the GLA-LSQ adjuvant, which is produced by IDRI.

## Code availability

No custom code was used in this study.
