# Supplementary material for: Immunofocusing humoral immunity potentiates the functional efficacy of the AnAPN1 malaria transmission-blocking vaccine antigen
Source: NPJ Vaccines. 2021 Apr 6;6:49. doi: 10.1038/s41541-021-00309-4 (PMC8024329; doi:10.1038/s41541-021-00309-4)
Supplement: Supplementary file 1 — Reporting Summary [file 41541_2021_309_MOESM1_ESM.pdf]

nature research

Corresponding author(s): Rhoel Dinglasan

Last updated by author(s): Feb 16, 2021

Reporting Summary

Nature Research wishes to improve the reproducibility of the work that we publish. This form provides structure for consistency and transparency in reporting. For further information on Nature Research policies, see our Editorial Policies and the Editorial Policy Checklist.

Statistics

For all statistical analyses, confirm that the following items are present in the figure legend, table legend, main text, or Methods section.

n/a	Confirmed	
☐	☒	The exact sample size (n) for each experimental group/condition, given as a discrete number and unit of measurement
☐	☒	A statement on whether measurements were taken from distinct samples or whether the same sample was measured repeatedly
☐	☒	The statistical test(s) used AND whether they are one- or two-sided<br>*Only common tests should be described solely by name; describe more complex techniques in the Methods section.*
☒	☐	A description of all covariates tested
☐	☒	A description of any assumptions or corrections, such as tests of normality and adjustment for multiple comparisons
☐	☒	A full description of the statistical parameters including central tendency (e.g. means) or other basic estimates (e.g. regression coefficient) AND variation (e.g. standard deviation) or associated estimates of uncertainty (e.g. confidence intervals)
☒	☐	For null hypothesis testing, the test statistic (e.g. F, t, r) with confidence intervals, effect sizes, degrees of freedom and P value noted<br>*Give P values as exact values whenever suitable.*
☒	☐	For Bayesian analysis, information on the choice of priors and Markov chain Monte Carlo settings
☒	☐	For hierarchical and complex designs, identification of the appropriate level for tests and full reporting of outcomes
☒	☐	Estimates of effect sizes (e.g. Cohen's d, Pearson's r), indicating how they were calculated

Our web collection on statistics for biologists contains articles on many of the points above.

Software and code

Policy information about availability of computer code

Data collection	No software was used for data collection.
Data analysis	Graphpad Prism 6 and R studio were used for data analysis. No custom code was used.

For manuscripts utilizing custom algorithms or software that are central to the research but not yet described in published literature, software must be made available to editors and reviewers. We strongly encourage code deposition in a community repository (e.g. GitHub). See the Nature Research guidelines for submitting code & software for further information.

Data

Policy information about availability of data

All manuscripts must include a data availability statement. This statement should provide the following information, where applicable:

- Accession codes, unique identifiers, or web links for publicly available datasets
- A list of figures that have associated raw data
- A description of any restrictions on data availability

The datasets generated during the current study are available on reasonable request from the corresponding author.

Field-specific reporting

Please select the one below that is the best fit for your research. If you are not sure, read the appropriate sections before making your selection.

☒ Life sciences ☐ Behavioural & social sciences ☐ Ecological, evolutionary & environmental sciences

For a reference copy of the document with all sections, see nature.com/documents/nr-reporting-summary-flat.pdf

Life sciences study design

All studies must disclose on these points even when the disclosure is negative.

Sample size	Since a single mouse cannot yield sufficient purified IgG for an assay, using current best practices, immune serum from either ten or twenty mice were pooled before purification. The purified IgG from the pooled immune sera permitted us to perform dose-response membrane feeding assays in triplicate.
Data exclusions	No data was excluded.
Replication	Immunization experiments were completed twice. All assays were performed with two-three technical replicates.
Randomization	Mice were randomly assigned to cages (by animal care staff) and cages were randomly assigned to treatment or cohort groups.
Blinding	Blinding is not routinely followed for membrane feeding assays for pre-clinical studies. Control groups are checked first to make sure that the laborious dissection and enumeration of midgut oocysts can proceed for all mosquito cups, since no infection in the control group indicates a failed experiment and all mosquitoes are killed and discarded. In human challenge studies for clinical trials for vaccines, wherein a larger staff size allows for it, a second reader who is blinded to treatment is utilized for enumerating oocysts. This manuscript reports on pre-clinical work.

Reporting for specific materials, systems and methods

We require information from authors about some types of materials, experimental systems and methods used in many studies. Here, indicate whether each material, system or method listed is relevant to your study. If you are not sure if a list item applies to your research, read the appropriate section before selecting a response.

Materials & experimental systems

n/a	Involved in the study	
☐	☒	Antibodies
☒	☐	Eukaryotic cell lines
☒	☐	Palaeontology and archaeology
☐	☒	Animals and other organisms
☐	☒	Human research participants
☒	☐	Clinical data
☒	☐	Dual use research of concern

Methods

n/a	Involved in the study	
☒	☐	ChIP-seq
☒	☐	Flow cytometry
☒	☐	MRI-based neuroimaging

Antibodies

Antibodies used	Antibodies used in ELISAs include (HRP)-conjugated goat anti-mouse IgG(H+L) (KPL) (Thermo Fisher Scientific #31430), Mouse Typer Isotyping Panel (Bio-Rad #1722055), and (HRP)-conjugated goat anti-rabbit IgG(H+L) (KPL) (Bio-Rad #1721019).
Validation	These antibodies have been independently verified by the manufacturer/vendor as per QA processes.

Animals and other organisms

Policy information about studies involving animals; ARRIVE guidelines recommended for reporting animal research

Laboratory animals	Female CD1 mice were used at 6-8 weeks of age.
Wild animals	No wild animals were used in the study.
Field-collected samples	The study did not included field-collected animal samples.
Ethics oversight	Use of vertebrate animals (mice) for these studies have been approved by the UF Animal Care and Use Committee (UF Animal Welfare Assurance number A3377-01, Protocol# 201909359, approved on 06/07/2019).

Note that full information on the approval of the study protocol must also be provided in the manuscript.

Human research participants

Policy information about studies involving human research participants

Population characteristics	Children 5-15 years of age (both genders) were screened for carriage of Plasmodium falciparum gametocytes. Informed consent from parent/guardian was sought and acquired before enrollment of the child in the studies. These are described in the methods section.
Recruitment	Participants were recruited based on the results of a microscopic examination of thin blood smears for gametocytes from each individual (see above characteristics).
Ethics oversight	All research protocols have been approved by the relevant institutional review boards and national ethics committees for Cameroon (2016/03/730 and 2019/07/1168/CE/CNERSH/SP) and the University of Florida (UF) for concurrence and laboratory-based studies (IRB201800971, IRB201902261 and IRB201602150).

Note that full information on the approval of the study protocol must also be provided in the manuscript.
