# Supplementary material for: Immunofocusing humoral immunity potentiates the functional efficacy of the AnAPN1 malaria transmission-blocking vaccine antigen
Source: NPJ Vaccines. 2021 Apr 6;6:49. doi: 10.1038/s41541-021-00309-4 (PMC8024329; doi:10.1038/s41541-021-00309-4)
Supplement: Supplementary file 2 — Supplemental Information [file 41541_2021_309_MOESM2_ESM.docx]

**Supplementary Materials**

**Supplementary Table 1. AnAPN1 v2.0 immunogen constructs.**

| **Construct Name / Amino Acid Sequence** | **pI / MW (Da)** |
| --- | --- |
| **Construct UF1:**  MCDERYRLPTTSIPIHYDLHLRTEIHRNERTFTGTVGIQLQVVQATDKLVMHNRGLVMSSAKVSSLPNGVTGAPTLIGDVQYSTDTTFEHITFTSPTILQPGTYLLEVAFQGRLATNDDGFYVSSYVADNGERRYLAHHHHHH | 6.39 / 16,105.05 |
| **Construct UF2:**  MCIQLQVVQATDKLVMHNRGLVMSSAKVSSLPNGVTGAPTLIGDVQYSTDTTFEHITFTSPTILQPGTYLLEVAFQGRLATNDDGFYVSSYVADNGERRYLAHHHHHH | 6.19 / 11,927.40 |
| **Construct UF3:**  MCDLHLRTEIHRNERTFTGTVGIQLQVVQATDKLVMHNRGLVMSSAKVSSLPNGVTGAPTLIGDVQYSTDTTFEHITFTSPTILQPGTYLLEVAFQGRLATNDDGFYVSSYVADNGERRYLAHHHHHH | 6.33 / 14,261.98 |
| **Construct UF4:**  MCAKFVAAWTLKAAADLHLRTEIHRNERTFTGTVGIQLQVVQATDKLVMHNRGLVMSSAKVSSLPNGVTGAPTLIGDVQYSTDTTFEHITFTSPTILQPGTYLLEVAFQGRLATNDDGFYVSSYVADNGERRYLAHHHHHH | 6.79 / 15,591.58 |
| **Construct UF5:**  MCDLHLRTEIHRNERTFTGTVGIQLQVVQATDKLVMHNRGLVMSSAKVSSLPNGVTGAPTLIGDVQYSTDTTFEHITFTSPTILQPGTYLLEVAFQGRLATNDDGFYVSSYVADNGERRYLAAKFVAAWTLKAAADLHLRTEIHRNERTFTGTVGIQLQVVQATDKLVMHNRGLVMSSAKVSSLPNGVTGAPTLIGDVQYSTDTTFEHITFTSPTILQPGTYLLEVAFQGRLATNDDGFYVSSYVADNGERRYLAHHHHHH  AKFVAAWTLKAAA (PADRE Linker) | 6.38 / 28,778.37 |
| **Construct UF6:**  MCDLHLRTEIHRNERTFTGTVGIQLQVVQATDKLVMHNRGLVMSSAKVSSLPNGVTGAPTLIGDVQYSTDTTFEHITFTSPTILQPGTYLLEVAFQGRLATNDDGFYVSSYVADNGERRYLAGSGGGGSGGGGSGGGGGSGDLHLRTEIHRNERTFTGTVGIQLQVVQATDKLVMHNRGLVMSSAKVSSLPNGVTGAPTLIGDVQYSTDTTFEHITFTSPTILQPGTYLLEVAFQGRLATNDDGFYVSSYVADNGERRYLACGGSGHHHHHH  GS[GGGGS]_2_GGGGSG (Glycine Linker) | 6.13 / 29,014.22 |
| **Construct UF6b:**  MCDLHLRTEIHRNERTFTGTVGIQLQVVQATDKLVMHNRGLVMSSAKVSSLPNGVTGAPTLIGDVQYSTDTTFEHITFTSPTILQPGTYLLEVAFQGRLATNDDGFYVSSYVADNGERRYLAGSGGGGSGGGGSGGGGGSGDLHLRTEIHRNERTFTGTVGIQLQVVQATDKLVMHNRGLVMSSAKVSSLPNGVTGAPTLIGDVQYSTDTTFEHITFTSPTILQPGTYLLEVAFQGRLATNDDGFYVSSYVADNGERRYLACGGSG​  GS[GGGGS]_2_GGGGSG (Glycine Linker) | 5.71 / 28,191.37 |

Calculated by <https://web.expasy.org/compute_pi/>

| **Analysis** | **Method** | **Acceptance Criteria** | **Results** | **Pass/Fail** |
| --- | --- | --- | --- | --- |
| Viable Cell Count: Pre-freeze | TM-0088 | ≥1x10^6^ CFU/mL | 3x10^8^ CFU/mL | Pass |
| Viable Cell Count: Post-freeze | TM-0088 | ≥1x10^6^ CFU/mL | 4x10^8^ CFU/mL | Pass |
| Lysogenic bacteriophage | 510088GMP.BUK (BioReliance) | Bacteriophage not detected | Not detected | Pass |
| Mycoplasma | 300201GMP.BUK (BioReliance) | Negative for Mycoplasma | Negative | Pass |
| Purity testing of microbial cell banks | 510008GMP.BUK (BioReliance) | No evidence of contaminating organisms | Absent | Pass |
| Morphology | 510057GMP.BUK  (BioReliance) | Gram negative, rod shaped bacterium | Conforms | Pass |
| Host Cell Phenotype | 512002GMP.BUK (BioReliance) | Profile consistent with host cell line | Conforms | Pass |
| Host Cell Genotype | 105058GMP.BUK (BioReliance) | Banding pattern of MCB consistent with host cell line | Conforms | Pass |
| Percentage of *E.coli* cells retaining expression vector | 104030GMP.BUK (BioReliance) | Report Results | 100% retain antibiotic resistance gene, 100% retain expression construct | N/A |
| Retention of the foreign UF6 gene: Sequencing | 106603GMP.BUK (BioReliance) | Conforms with the reference sequence for the coding sequence | Conforms | Pass |
| Plasmid Identity: Restriction Digestion | 104034GMP.BUK (BioReliance) | Coherent fragments with plasmid restriction map | Conforms | Pass |
| Retention of the recombinant construct – determination of plasmid copy number | 107315GMP.BUK (BioReliance) | Report Results | Test article AF85BF contained between 21.81 and 87.22 copies of AF85BF transgene target per cell  (43.61 ± 2-fold) | N/A |
| Expression of ΔN-AnAPN1 (UF6) | TM-0087 | Anti-UF6b antisera identified a protein of MW 28 kDa | Conforms to Reference^1^ | Pass |

**Supplementary Table 2. UF6b-phoA-T7 Master Cell Bank (MCB)**

^1: Reference EVENT-2020-0039^

**Supplementary Table 3. Certificate of Testing for UF6b Drug Product.**

| **Analysis** | **Method** | **Results** |
| --- | --- | --- |
| pH | USP <791> | 8.05 |
| Visual Appearance | Visual Inspection | Free from visible particulates, slightly opalescence, slightly yellow |
| Identity | Western Blot | Conforms to Major Band in Reference Standard |
| Protein Concentration | Spectrophotometric/ A280 | 3.96 mg/mL |
| Purity | SEC-HPLC | Four peaks, in sequence low MW to high MW^c^  Peak 1: 48.04%  Peak 2: 33.09%  Peak 3: 10.91%  Peak 4: 7.96% |
| Purity | RP-HPLC | 100% |
| Purity | CE-SDS-PAGE | 96.43% |
| Residual DNA | PicoGreen | 24.3 ng/mL |
| Residual HCP | ELISA | 206 ng/mg |
| Endotoxin | USP<1115> | 6.7 EU/mg |
| Bioburden | USP<85> | TAMC 0 cfu/mL  TYMC 0 cfu/mL |

^a^TAMC, Total Aerobic Microbial Count

^b^TYMC, Total combined yeasts and molds count

^c^Refer to Supplementary Figure 5.

**Supplementary Table 4. Immunization groups.**

| **Experimental Groups** | **Dose** | **Number of mice/groups** | **Volume of UF6b-stock (0.4 mg/mL)** | **Volume of Adjuvant** |
| --- | --- | --- | --- | --- |
| **A**: UF6b alone | 20 µg | 10 | 0.6 mL | (0.6 mL diluent buffer instead of adjuvant) |
| **B**: UF6b:GLA-LSQ | 20 µg | 20 | 1.2 mL | 1.2 mL |
| **C**: UF6b:AddaVax™ | 20 µg | 10 | 0.6 mL | 0.6 mL |


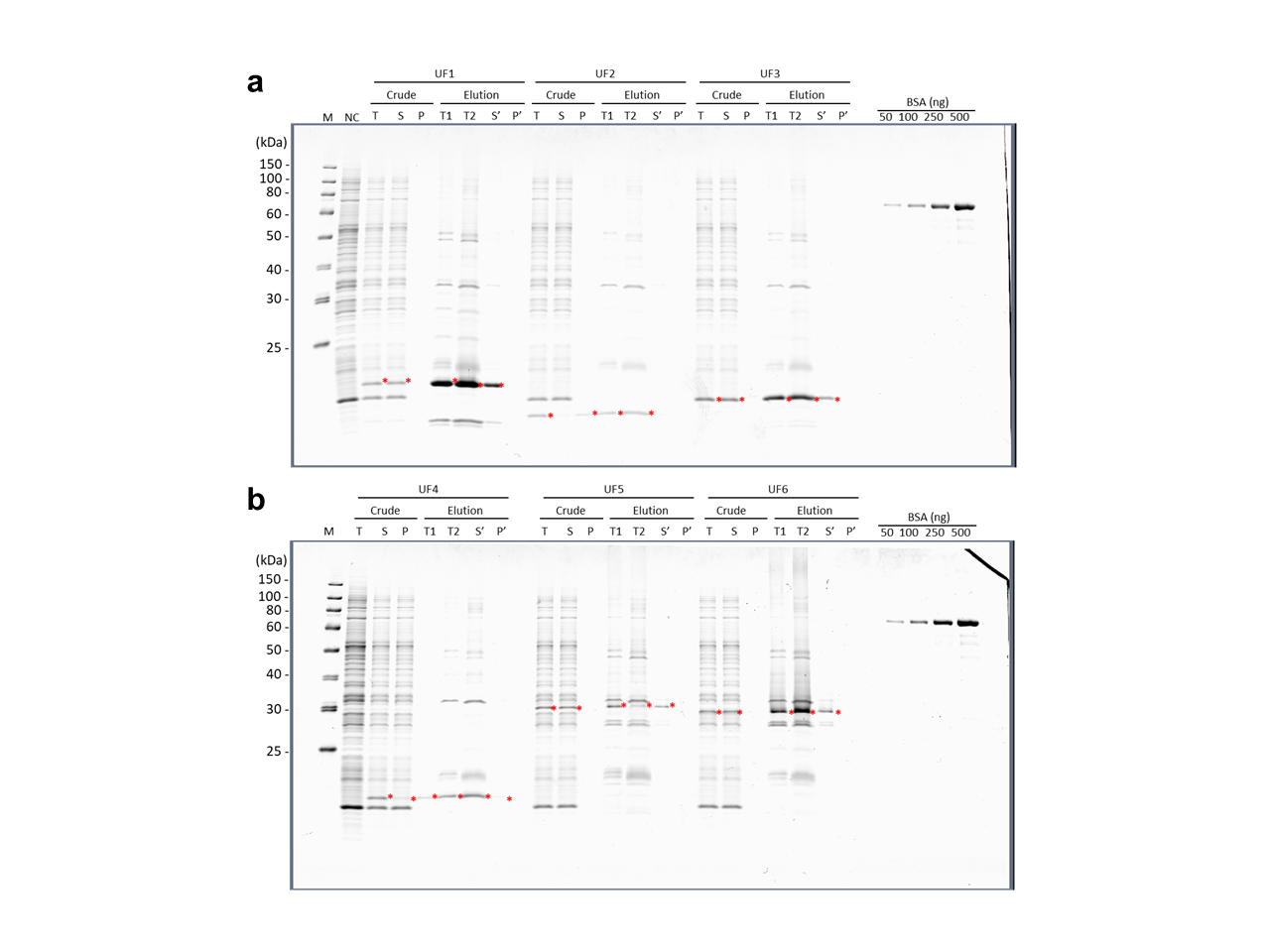


**Supplementary Figure 1. Expression and purification test for proteins UF1 to UF6 using a wheat germ cell-free protein expression system.** Proteins UF1-UF3 **(a)** and UF4-UF6 **(b)** were expressed for 72 h in 50 µL translation reactions using a wheat germ cell-free protein expression system followed by purification on a Ni resin using a His-tag at the C-terminus of the proteins and isolated after centrifugation as further outlined in the Methods. All blots derive from the same experiment and were processed in parallel and the entirety of each blot is shown. All fractions were analyzed on 15% SDS-PAGE along with a BSA standard: M = Molecular weight marker (complete set) as indicated in the figures; T = Total fraction from crude translation reaction mixture; S = Soluble fraction taken from crude translation reaction mixture after centrifugation; P = Pellet fraction taken from crude translation reaction mixture after centrifugation; T1 = Elution fraction 1; Elution fraction 2; S’ = Soluble fraction of elution fractions after centrifugation; P’ = Pellet fraction taken from elution fractions after centrifugation; BSA = reference standard; ***** = indicating expressed proteins.





**Supplementary Figure 2. Initial immunogenicity screen of GLA-LSQ and Alhydrogel™ formulations with UF3, UF4, UF5 or UF6 as compared to UF1. (a)** Pre-immune sera and (**b)** Day 56 sera (N=5 mice) for wheat germ cell-free expressed antigens (20μg doses) formulated with GLA-LSQ adjuvant. **(c)** Pre-immune sera and (**d**) Day 56 sera (N=5 mice) for antigens (20μg doses) formulated with Alhydrogel (ALUM) adjuvant. Error bars indicate SEM of triplicates.

**
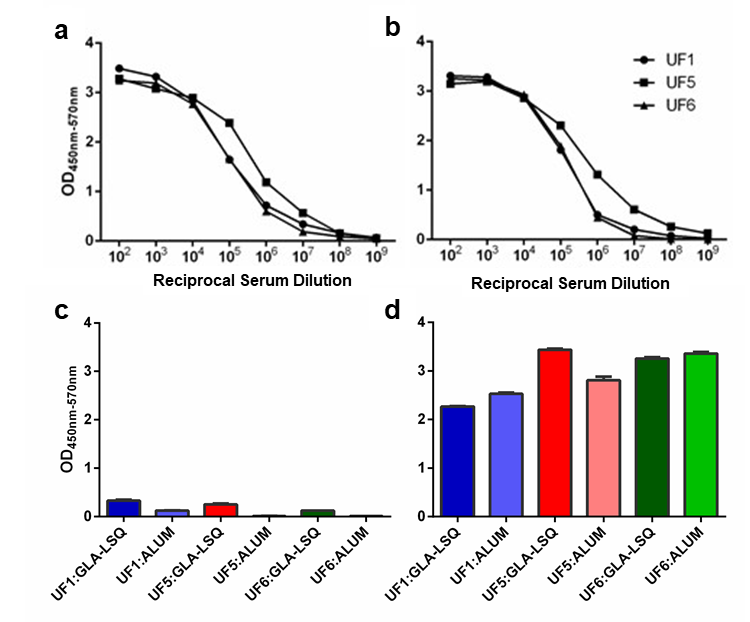
**

**Supplementary Figure 3. Comparison of GLA-LSQ and Alhydrogel™ formulations with UF1, UF5 and UF6. (a-b)** Anti-UF1, UF5, and UF6 antibody titers from mice immunized with 20 µg/mL of antigen were determined by indirect ELISA. The antigen was formulated with GLA-LSQ (**a)** or Alhydrogel**™** (indicated as ALUM) (**b**). Anti-Peptide 7 titers were determined by indirect peptide ELISA with sera diluted 1/1,000 **(c)** Anti-Peptide 9 titers were determined by indirect peptide ELISA with sera diluted 1/1,000 **(d)**. Error bars indicate SEM of triplicates.


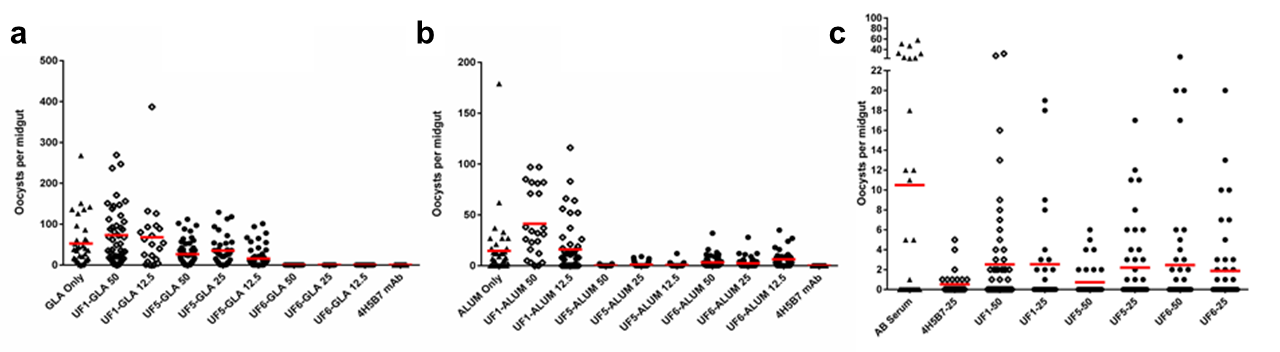


**Supplementary Figure 4. Screening Study: Functional transmission-activity assessment.** Direct Membrane Feeding Assay (DMFA) conducted with UF1, UF5, and UF6 either with GLA-LSQ **(a)** or Alhydrogel™ (indicated as ALUM) **(b)** performed using infectious, gametocytemic blood from a single carrier during the May-July transmission season in Cameroon. Antibody concentrations (ranging from 12.5-50 µg/mL) per feeder are indicated. **(c)** Representative SMFA with UF1, UF5, and UF6 formulated with GLA-LSQ. 4H5B7 is a monoclonal antibody (mAb) to peptide 7, which serves as a positive control. In panel A-B, 4H5B7 was used at a single concentration of 50 µg/mL, while in panel C, 25 µg/mL was used. Mean oocyst number/mosquito midgut is shown by the red horizontal bar.

**
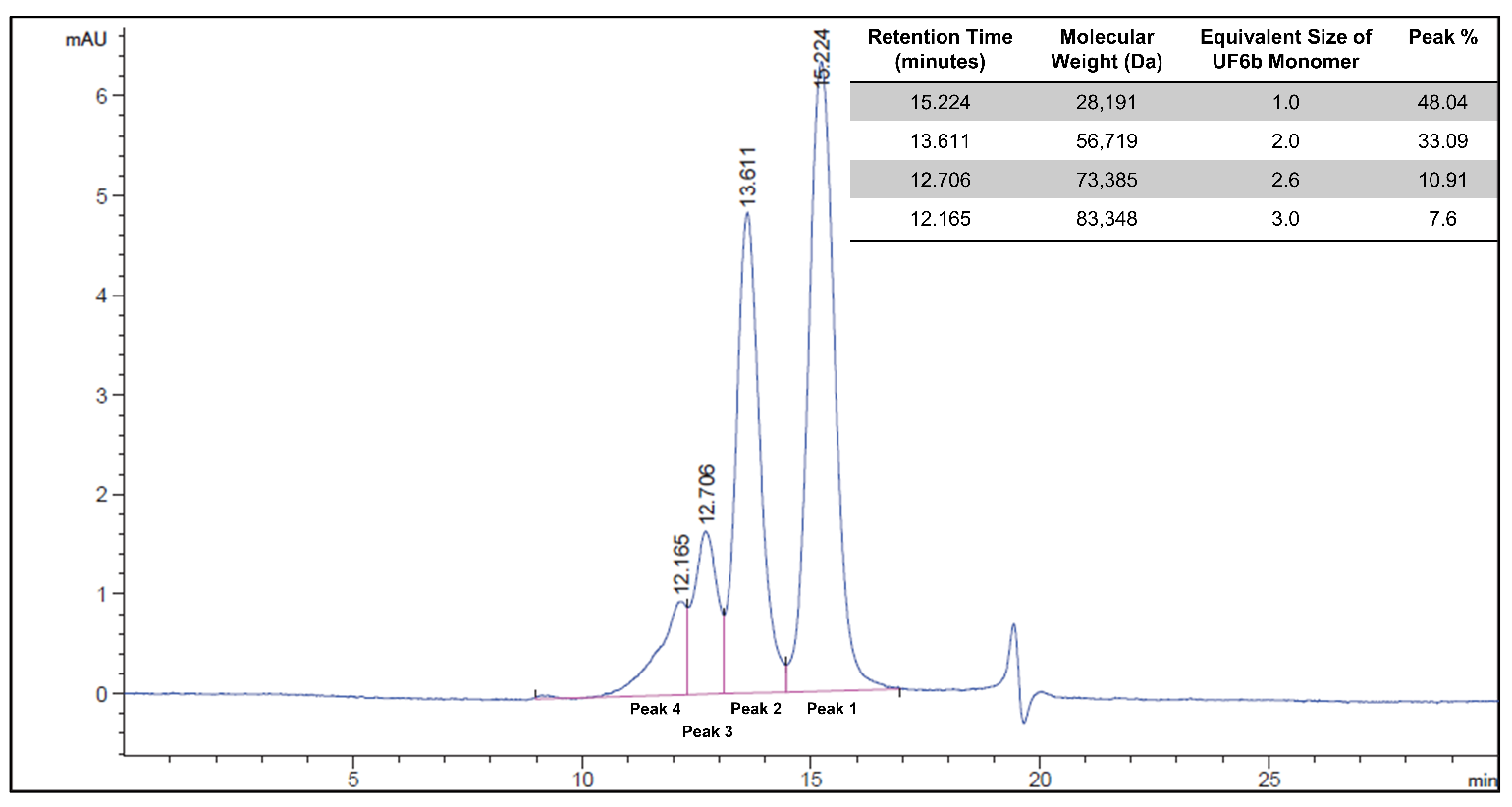
**

**Supplementary Figure 5. Size-exclusion chromatography high performance liquid chromatography (SEC-HPLC) analysis of UF6b described in Supplementary Table S3.** SEC-HPLC was performed under non-reducing, non-denaturing conditions allowing for formation of higher order oligomerization. The four peaks correspond to retention times of 15.224 minutes (48%), 13.611 minutes (33%), 12.706 minutes (11%), and 12.165 minutes (8%). Monomeric UF6b exhibited a retention time of 15.224 minutes with molecular mass of 28,191 Daltons. The faster retention times had molecular masses of 56,719 Da (13.611 minutes), 73,385 Da (12.706 minutes), and 83,348 Da (12.165 minutes). These correspond to monomer (15.224 minutes), dimer (13.611 minutes), trimer (12.165 minutes), and likely a conformational alteration of the dimeric form due to reduced intramolecular interactions (12.706 minutes).


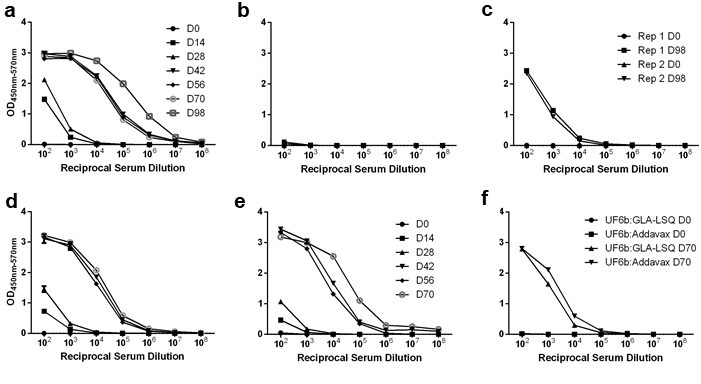


**Supplementary Figure 6. Indirect enzyme-linked immunosorbent assay results of replicate mouse studies. (a-c)** A second cohort of mice were immunized (i.m.) with UF6b:GLA-LSQ in a prime and two boost regimen (boosts on D28 and D70) for the 98-Day study (20 μg/dose/mouse). Antibody response to UF6b **(a),** peptide 7 **(b),** and peptide 9 **(c)** were measured. The peptide 9-specific response in the second cohort is plotted along with that from the first cohort of mice tested (Indicated as Rep 2 and Rep 1, respectively). **(d-f)** The 70-Day study was also repeated in which mice were immunized in a prime and two boost regimen (boosts on D28 and D56) (20 μg/dose/mouse) with either UF6b:GLA-LSQ **(d)** or UF6b: AddaVax™ **(e).** The antibody responses to UF6b **(d-e)** and peptide 9 **(f)** were measured. ELISAs were performed on pooled sera collected at designated timepoints. Error bars indicate SEM of triplicates.


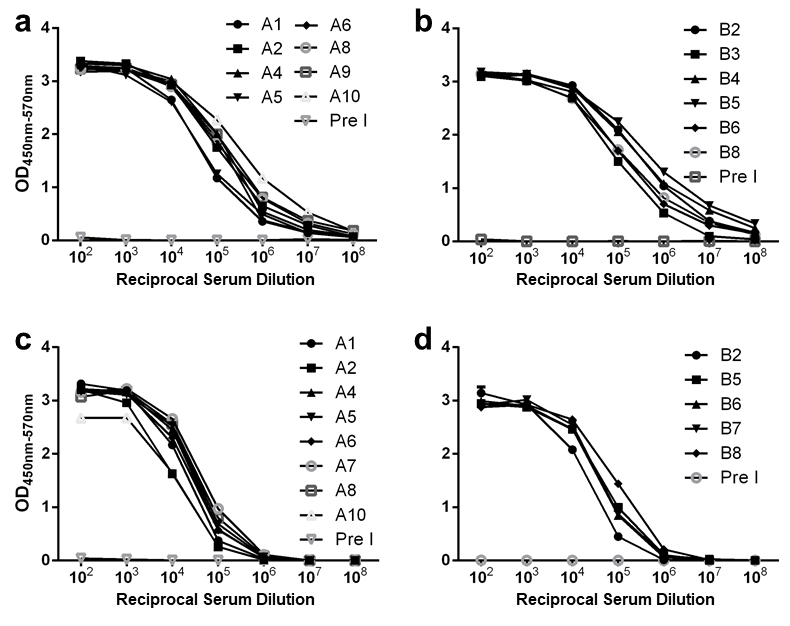


**Supplementary Figure 7. Indirect enzyme-linked immunosorbent assay results for individual mice.**

**(a-d)** Mice were immunized in a prime and two boost regimen (boosts on D28 and D56) (20 μg/dose/mouse) for the 70-Day study with either UF6b:GLA-LSQ **(a,c)** or UF6b:AddaVax™ (**b,d)** in two separate cohorts of mice **(a,b** and **c,d)**. A1-10, B1-8: mouse identification numbers. Each data point represents an individual mouse. Error bars indicate SEM of triplicates.
